# Design and Deployment of Low-Cost Plastic Optical Fiber Sensors for Gas Monitoring

**DOI:** 10.3390/s150100485

**Published:** 2014-12-30

**Authors:** Sabrina Grassini, Maen Ishtaiwi, Marco Parvis, Alberto Vallan

**Affiliations:** 1 Department of Applied Science and Technology, Politecnico di Torino, Corso Duca degli Abruzzi, 24 Torino, Italy; 2 Department of Electronic and Telecommunications, Politecnico di Torino, Corso Duca degli Abruzzi, 24 Torino, Italy; E-Mails: maen.ishtaiwi@polito.it (M.I.); marco.parvis@polito.it (M.P.); alberto.vallan@polito.it (A.V.)

**Keywords:** gas sensors, plasma deposition, PECDV, plastic optical fibers

## Abstract

This paper describes an approach to develop and deploy low-cost plastic optical fiber sensors suitable for measuring low concentrations of pollutants in the atmosphere. The sensors are designed by depositing onto the exposed core of a plastic fiber thin films of sensitive compounds via either plasma sputtering or via plasma-enhanced chemical vapor deposition (PECVD). The interaction between the deposited layer and the gas alters the fiber's capability to transmit the light, so that the sensor can simply be realized with a few centimeters of fiber, an LED and a photodiode. Sensors arranged in this way exhibit several advantages in comparison to electrochemical and optical conventional sensors; in particular, they have an extremely low cost and can be easily designed to have an integral, *i.e.*, cumulative, response. The paper describes the sensor design, the preparation procedure and two examples of sensor prototypes that exploit a cumulative response. One sensor is designed for monitoring indoor atmospheres for cultural heritage applications and the other for detecting the presence of particular gas species inside the RPC (resistive plate chamber) muon detector of the Compact Muon Solenoid (CMS) experiment at CERN in Geneva.

## 1. Introduction

Fiber optic sensors are being employed for various sensing applications, e.g., optical fiber sensors are used for detecting the presence of air pollutants in the atmosphere [1,2], as well as being employed in the biomedical field [3,4] and in environmental sensing [5,6]. This wide diffusion is mainly due to the relevant advantages of optical fibers, such as the intrinsic fire-safe behavior and the immunity to electromagnetic interference (EMI), which make them suitable for *in situ* measurements, also in adverse environments.

The most common approach to obtain a fiber optic sensor (FOS) is to use a glass fiber, whose core is modified to have a periodic change of its refractive index, thus creating the so-called fiber Bragg grating (FBG). FBG sensors can be employed to arrange intrinsic strain and temperature sensors and are widely used, in conjunction with assemblies that produce controlled fiber strain, to measure different kinds of quantities. The main problem of this kind of sensor is the cost of the interrogation system, which must be capable of selectively measuring at specific wavelengths. In addition, the small diameter of typical glass fibers requires special attention in their illumination and the use of low-tolerance, high-cost connectors.

Plastic optical fibers (POF), in comparison, have a lower cost per meter and usually a higher diameter that results in relaxed specifications on the connectors and in easy illumination. On the other hand, POF have a higher attenuation with respect to glass fibers; this limits their use in the transmission field, but is usually not a problem for sensor development, so that POF use in this field is continuously increasing [7].

POF can be used to arrange sensors by using most of the approaches already employed in glass fibers. FBG, both normal and long-period, can be inscribed into low-cost plastic optical fiber to obtain different kinds of sensors [8], but the problem of the interrogation system cost still may limit the possible applications. Similarly, other proposed solutions, such as backscattering, optical time-domain reflectometers, fluorescence measurements [9] and selective excitation of surface plasma waves in the so-called surface plasmon resonance [10], even though capable of providing quite interesting results, usually require complex and costly setups.

On the other end, solutions based on the fiber light attenuation measurements could be extremely cheap and simple [11], even though at the expense of reduced stability, as the fiber light transmission capability is affected by a wide number of parameters.

By measuring the fiber light attenuation, it is very easy to directly arrange bending and pressure sensors, but the measurement of chemical quantities requires a chemical modification of the fiber core surface in order to make it reactive with respect to different chemical species with high sensitivity and selectivity [7].

This latter solution has great potentialities, and it can be employed for many different quantities simply by changing the nature of the sensitive coating. Sensors have been developed for measuring the pH of liquid solutions and for detecting the presence of several chemicals, such as methanol, ethanol, toluene and other gases in the air [7]. All of these sensors are designed to detect the amount of chemical present, but the use of specific coatings opens the possibility of designing sensors having a cumulative response, *i.e.*, of devices whose output is connected with the total exposition of the sensor to a specific quantity. Designing sensors with this behavior requires the development of coatings that are affected by non-reversible chemical reactions, but that enable the creation of sensors capable of detecting the presence of extremely low concentrations of specific pollutants

This paper, therefore, describes a procedure that has been successfully employed by the authors to arrange simple and inexpensive plastic optic-based sensors that exploit a cumulative response. These sensors are designed to detect chemicals in the gas phase and to exploit the interaction of the chemicals with the evanescent electromagnetic field at the core surface. The sensor assemblies employ a light amplitude measurement, which requires only an LED and a photo-diode and are arranged by taking advantage of vacuum deposition techniques to make the fiber sensitive to the quantity of interest.

## 2. Plastic Optical Fiber Sensors

As discussed in the previous section, in order to deploy a fiber optic-based sensor whose transmittance is affected by the materials surrounding the fiber itself, two operations have to be performed.

**Figure 1.**
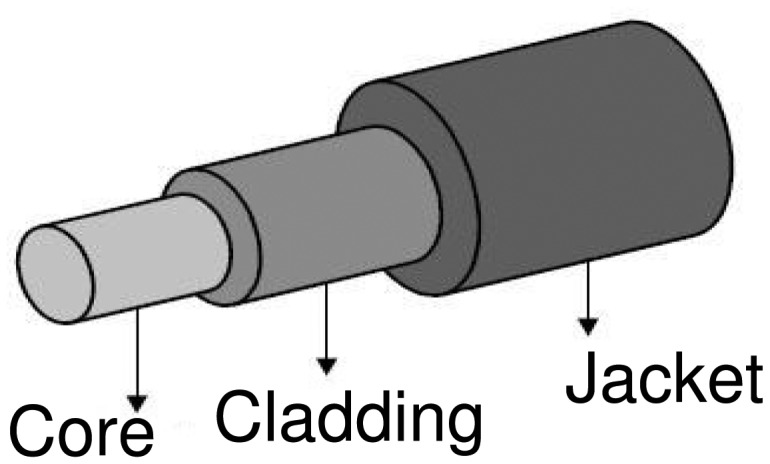
Plastic optical fiber structure (not to scale).

Firstly, the fiber core must be exposed to place the evanescent field in contact with the surroundings. This is done by removing the fiber jacket and then by removing the fiber cladding, thus exposing the fiber core (Figure 1).

**Figure 2.**
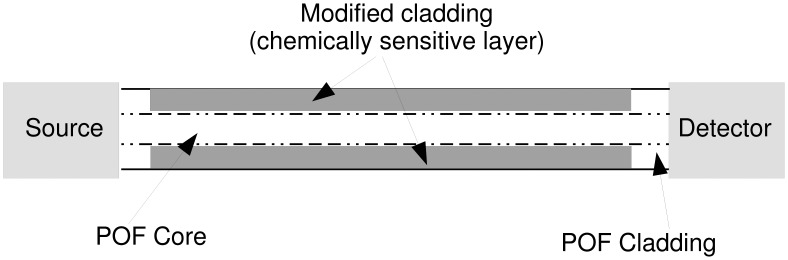
Configuration of the plastic optical fiber sensor.

Secondly, a sensitive material, often referred to as modified cladding, has to be chosen and deposed onto the core in such a way that the presence of the substance to be detected is capable of changing the fiber optical properties, thus affecting the fiber transmittance (Figure 2).

Several kinds of plastic fibers can be found on the market; however, the most common and inexpensive fibers are the ones designed for short-range telecommunication connections, which have costs of the order of 1€per meter. These fibers are highly multi-mode with a typical diameter of 1 mm, with a poly-methyl-methacrylate (PMMA) core diameter of about 980 *μ*m and a fluoropolymeric cladding 10 *μ*m thick.

The first step is therefore the removal of the fluoropolymeric cladding, which has to be obtained without damaging the core, a condition that could easily lead to unacceptable transmission losses and or a fragile core. Unfortunately, the cladding has mechanical properties quite similar to the core, so a mechanical approach is usually unsuitable, as it is difficult to discriminate when the cladding removal is complete.

A chemical approach, by using a solvent capable of attacking the cladding without damaging the core, is a viable alternative, since the cladding of most POF is composed of a fluoropolymer, while the core is PMMA. As an example, Merchant [12] describes a solution based on the use of acetone, which has been successfully applied to uncover the core. The authors instead decided to use an approach based on ethyl-acetate, which can be easily applied also in the absence of specific tools. The procedure consists of dipping the fiber in ethyl-acetate until the solvent breaks the bonds between the cladding and the PMMA, so that the cladding can be easily rubbed away by using a simple tissue-paper without damaging the core.

The exposure time is critical, since too long of a dipping in the solvent can damage the PMMA core; however, consistent results in the case of typical fibers with a 1 mm diameter can be obtained by using 99.5% ethyl-acetate for 40 s at a temperature of about 25 °C.

The effectiveness of the cladding removal can be assessed by using a field emission scanning electron microscope (FESEM) to observe the core.

**Figure 3.**
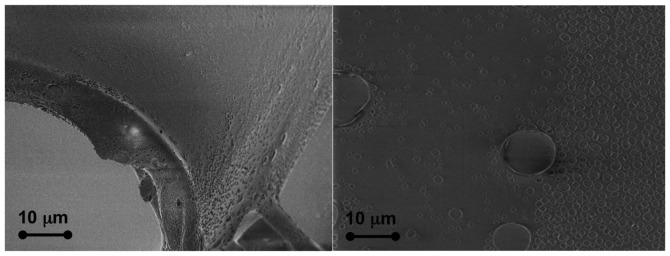
The effect of the ethyl-acetate procedure on the fiber surface. (**Left**) The point where the cladding is removed: the 10 *μ*m edge is clearly visible; (**Right**) The core surface after the removal procedure: the core appears clean and undamaged.

Figure 3 shows two images obtained with a Supra 40 Zeiss FESEM equipped with an energy dispersive X-ray spectrometer (EDS) for elemental analysis. The pictures show how the proposed procedure is able to completely remove the cladding. The PMMA core presents only a few small defects with a size of less than 100 nm and a thickness of a few nanometers without any other visible damage.

Once the core is exposed, it is possible to coat it by using several different techniques, depending on the composition of the modified cladding, with the only limitation connected with the limited thermal resistance of the plastic fiber. If, as usual, the fiber is made of PMMA, the maximum temperature is typically limited to 70 °C. Among the different techniques, sol gel [13] has been used for the deposition of thick coatings, which are sensitive to pH and other quantities mainly in the liquid phase, while vacuum techniques, such as DC plasma sputtering and plasma-enhanced chemical vapor deposition (PECVD) [14] have been used for making sensors sensitive to gas compounds.

Sections 3 and 4 describe two examples of procedures for the development of gas sensors, which are based on DC plasma sputtering and PECVD, but, as described above, other procedures can be used, as well.

Basically, two approaches can be followed to select the coating material. One possibility is to use a material that initially reflects the light, *i.e.*, which does not adsorb the evanescent field and which becomes less reflective in the presence of the substance to be detected. This approach results in a sensor that has a decreasing transmittance when the substance to be detected is present. On the other hand, if the modified cladding makes the fiber lossy, usually as a consequence of a high refractive index that decreases when the substance to be detected is present, the sensor has an increasing transmittance when the substance is present. In the following sections, two examples referring to the two types of sensors will be described along with their deployment and characterization.

Eventually, the sensitized fiber is interrogated by using a light source and a light-sensing device and has to be mounted, so that its mechanical configuration remains stable. This last point is extremely important, since the transmittance of highly multi-mode fibers strongly depends on fiber bending [15,16].

**Figure 4.**
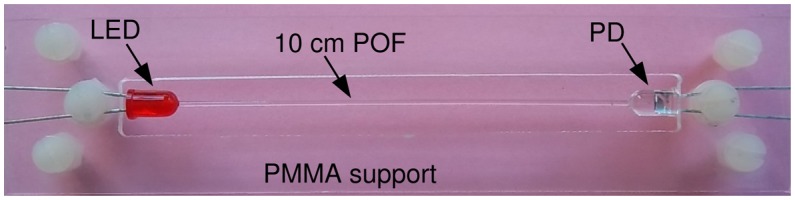
Sensor assembly composed of fiber, LED and photodiode.

To this aim, an arrangement, like the one shown in Figure 4, can be used. The assembly is composed of a piece of fiber with a length of the order of 10 cm, an LED, a photodiode and a PMMA support, which holds the fiber and diodes. Both the LED and photodiode have the conventional 5 mm diameter case. These semiconductors are prepared by drilling a 1 mm hole through their top: a PMMA glue is used to bond the fiber, whose ends are inserted into the holes, to the diodes. The assembly is then cured by putting it into an oven at 60 °C for four hours to polymerize the glue. Eventually, the assembly is fixed onto the support.

By using this assembly, most of the problems connected with either mechanical bending or thermal dilatation are made negligible, since the thermal expansion coefficients of both the fiber and support are quite similar. In addition, the mounting procedure with plastic screws allows one to easily mount and unmount the fiber and, therefore, to coat the core, either before or after having it mounted on the holder. After the sensor is arranged, *i.e.*, after the fiber core is coated with the sensitive material, the photodiode has to be painted, to avoid capturing external light.

Figure 4 shows the use of a red LED, which has been selected in this prototype according to the considerations expressed in Section 4, but other LED colors can be used, as well, depending on the specific behavior of the used coating.

## 3. H_2_S Sensor Obtained via DC Plasma Sputtering Example

This first example describes a solution designed for the cultural heritage field [17], where old wood showcases can produce small amounts of hydrogen sulfide (H_2_S) and of formic acid (HCOOH). Both are usually generated in less than a part-per-million concentration, thus being hardly detectable by conventional sensors, but the exposition of metallic artifacts, e.g., silver jewels and coins, to them for a long time leads to tarnishing and impairs their conservation and aspect, so that it should be avoided. A fiber-based sensor with a cumulative response in this case can act as an extremely sensitive device capable of detecting the overall exposure to the gas compounds instead of the gas concentration, which has a very low value. This behavior can be easily obtained by employing silver as the sensitive layer. The gas reaction of silver with hydrogen sulfide is:

(1) $$2{\text{Ag}}^{+}+{\text{S}}^{--}\Rightarrow {\text{Ag}}_{2}\text{S}+2{\text{e}}^{-}$$

Such a reaction is not reversible and has a first-order law kinetic with respect to the sulfide concentration *S*_C_:

(2) $$-\frac{dS_{Ag}}{dt}=k_{\text{S}}S_{\text{C}}$$

where *k*_S_ is the kinetic constant that mainly depends on the temperature and *S*_C_ is the sulfide concentration.

The effect of this tarnishing reaction is that the silver surface loses its brilliance with time, becoming darker. The idea is therefore to coat the fiber core with a very thin layer of silver, so that any tarnishing reaction occurs very quickly and to observe the transmittance reduction due to the silver reflectivity loss as the metallic silver turns into silver sulfide.

**Figure 5.**
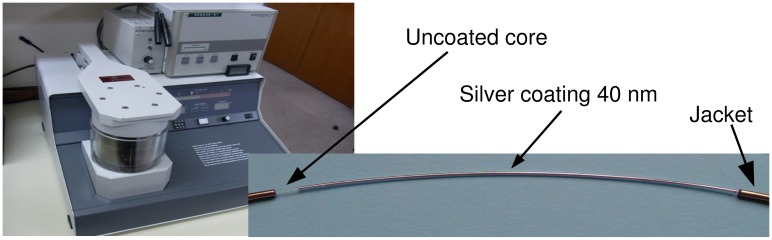
DC sputter used for the deposition (**left**) and an example of a fiber coated with 40 nm of silver (**right**).

Since silver is metallic, a simple DC sputtering, like the one shown in Figure 5, can be used to deposit it onto the fiber.

The sensing film must have a thickness that is enough to interact with the evanescent field, but small enough to be quickly affected by the tarnishing reaction.

This way, the tarnishing reaction, which is non-reversible, is able to produce measurable propagation losses when the sensor exposed is to the sulfide vapors. After some preliminary tests, a value of the order of 40 nm was found to provide the best values. The DC plasma reactor employed an Ag target (purity 99.99%) and was run by using Ar (purity 99.99%) as the discharge gas; the pressure was taken to a few Pascals by using a turbo-molecular pump in order to obtain a nanostructured silver layer.

Figure 5 also shows a picture of a fiber coated with a 40 nm-thick silver layer, which is used to arrange the sensor assembly as described in previous section.

**Figure 6.**
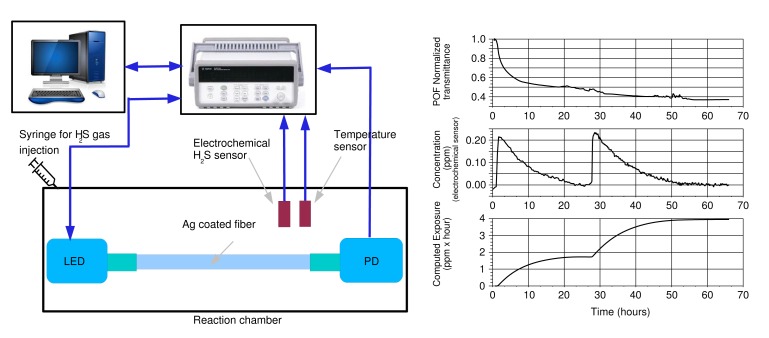
Setup used for characterizing the H_2_S sensor (**left**) and an example of traces obtained during the test (**right**).

Since the sensor is designed to detect very low concentrations of H_2_S, a direct characterization of it by using a standard sensor is quite difficult to obtain, and in addition, it is also difficult to provide stable and very low concentrations of H_2_S vapor. The solution adopted by the authors therefore is a characterization carried out by putting the sensor inside a reaction chamber, where, from time to time, a small amount of gas containing H_2_S is injected. The chamber is not sealed on purpose, so that, after a certain time, the air circulation removes all of the H_2_S, back to clean air. Figure 6 shows the proposed system, which employs a standard electrochemical H_2_S sensor designed for a range of 50 ppm as the reference measuring system.

In order to obtain meaningful measurements in the presence of very small H_2_S concentrations, the zero level of the reference sensor is compensated for just before each injection, when the air is known to be free from H_2_S. A Na_2_S 0.01 M solution is left in equilibrium with air inside an auxiliary bowl, and part of the air containing the H_S_ vapors is taken by using a syringe and injected into the reaction chamber to obtain peak concentrations of less than 0.5 ppm. Figure 6 shows on the right an example of traces recorded by the POF sensor and by the electrochemical sensor. On the bottom of Figure 6 is the exposure, *i.e.*, the integral of the electrochemical sensor output numerically computed starting from its values. In the example, it is clear how the POF sensor of this test saturates after the first injection, *i.e.*, with an exposure of less than 2 ppm · hour.

**Figure 7.**
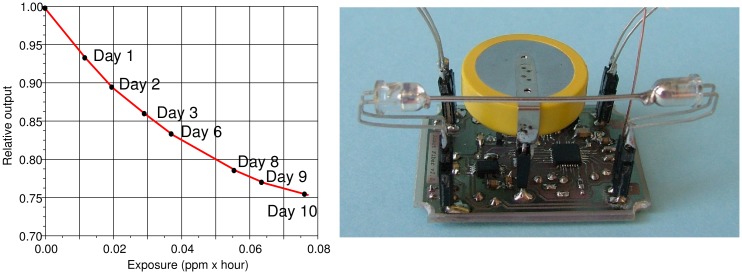
(**Left**) The sensor response to H_2_S exposure obtained according to the procedure previously described: each dot represents the transmittance value recorded at the end of each day after an H_2_S gas injection; (**Right**) An example of the setup composed of the fiber-based sensor and of the wireless communication system.

Figure 7 shows the response of a typical POF sensor during a test performed over a long time interval and with H_2_S peak concentrations ten-times lower than the ones used for obtaining the traces of Figure 6. The transmittance decreases with an exponential slope as expected from the reaction kinetics, and the sensor reduces its output of about 25% after an exposure to less than 0.1 ppm · hour. In the described test, the average H_2_S concentration is about 0.4 ppb. The same figure, on the right, shows one of the arrangements deployed for the sensor, which couples the POF assembly with a battery-operated wireless transmitter and that can be installed inside a showcase without the necessity of any intervention on its topology.

## 4. HF Sensor Obtained via PECVD Deposition

This second example describes the deployment of a sensor that exploits an increasing transmittance when exposed to the quantity to be detected. The capability of the fiber to transmit the light is related to differences of refractive indexes between the core and the material deposited onto the core. If a material with a higher refractive index is deposited, the fiber loses part of its capability to transmit the light, and any degradation of this high refractive index coating results in an increase of the transmitted light. This approach has been followed to arrange sensors with the capability of detecting low concentrations of HF vapors.

The sensors described in this section [18,19] have been designed to monitor the concentration of hydrogen fluoride vapors inside the resistive plate chamber (RPC) detectors of the Compact Muon Solenoid experiment at CERN laboratory in Geneva (Switzerland) [20]. These RPCs are composed of two Bakelite plates, maintained at a distance of 2 mm. An electric field of about 4.5 kV/mm is maintained between the plates and an insulating gas mixture composed of C_2_H_2_F_4_, C_4_H_10_ and % SF_6_ flows between the plates. The ionization of this gas upon the passage of a charged particle is the key element of these detectors.

Unfortunately, during the RPC use, the gas mixture gets contaminated due to the production of small amounts of HF, which increases the quiescent RPC current and reduces their detection capability [21]. For this reason, the gas mixture needs to be continuously filtered to remove the HF. The monitoring of the amount of HF to which the gas filters have been exposed is important to avoid filter degradation and to schedule their maintenance.

The material that is unaffected by most compounds and that gets corroded almost only by the HF is the glass. For this reason, to detect the fluoride ions originating from the dissociation of the gaseous HF, the authors decided to employ a glass-like thin film deposited onto the fiber core. SiO_2_-like films have a refractive index higher than PMMA; thus, a film of this type on the core makes the fiber quite lossy. In addition, glass-like films are very stable and almost unaffected by quantities different from HF, so that the sensor is extremely selective. Eventually, the reaction between glass and fluoride ions, which leads to the formation of silicon tetrafluoride (SiF_4_):

(3) $${\text{SiO}}_{2}+4\text{HF}\Rightarrow {\text{SiF}}_{4}+2{\text{H}}_{2}\text{O}$$

is not reversible; thus, the sensor is inherently suitable for measuring the total exposure to fluoride ions and to estimate the gas filter intoxication without the necessity of numerical post processing.

**Figure 8.**
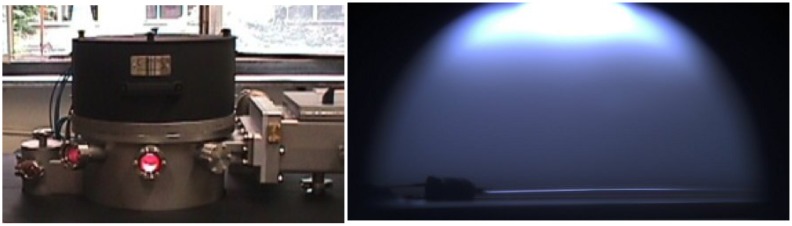
(**Left**) A picture of the PECVD reactor used for the deposition of the SiO_2_-like film; (**Right**) An image of the plasma discharge and of the fiber during the deposition.

A stoichiometric SiO_2_ glass layer cannot be easily obtained at low temperatures; however, SiO_x_ thin films can easily be obtained by plasma-enhanced chemical vapor deposition (PECVD) starting from organosilicon monomers. Figure 8 shows a picture of the reactor used by the author for the deposition along with an image of the plasma glow discharge and of the fiber during the deposition.

The deposition was obtained by using a discharge gas composed of tetraethoxysilane (TEOS) along with O_2_ and Ar in different ratios, at a pressure of 5 Pa and with an input power of 50 W. The amount of O_2_ in the gas feeding the plasma reactor chamber plays a critical role in the deposition, since a plasma rich in oxygen produces films that are almost inorganic, *i.e.*, with a composition close to SiO_2_, while a reduced amount of oxygen leads to organic SiO_x_ films. The former have a higher refractive index, but are more fragile, while the latter have a lower refractive index, but are much more conformable.

The film thickness can be controlled by varying the deposition time. The maximum sensor sensitivity is obtained with a film thick enough to make the fiber lossy, but thin enough to be quickly attacked by the fluoride ions. After some tests, a value of about 200 nm, which was obtained by using depositions times of about 15 min, was found to be a satisfactory compromise. The thickness of the film was measured by means of an FESEM.

Two different gas compositions, one without oxygen (*i.e.*, with the composition TEOS : O_2_ : Ar = 1 : 0 : 30) and one rich in oxygen (*i.e.*, with the composition TEOS : O_2_ : Ar = 1 : 20 : 10), were used to obtain two different film types and test for the effect of film composition.

**Figure 9.**
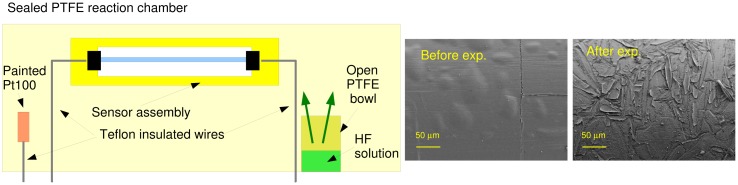
(**Left**) Setup used for sensor characterization; (**Right**) FESEM images of the fiber surface aspect before and after exposure to the HF vapors.

The sensor characterization requires exposing the fiber to an atmosphere containing a known amount of hydrogen fluoride. Even though it is possible to find gas cylinders containing pure hydrogen fluoride gas, the mixing of a known amount of gas with air in order to obtain vary low HF concentrations is quite difficult, so that the author employed the approach that is depicted in Figure 9.

The fiber sensor, along with a temperature sensor, was inserted into a sealed Polytetrafluoroethylene (PTFE) bowl, where also a small bowl containing an aqueous solution of HF acid was inserted. The HF liquid solution evaporates and comes into equilibrium with the air of the sealed reaction chamber. The HF vapor concentration, which is a function of temperature and HF concentration in the solution, can be computed by means of the well-known Antoine law [22]:

(4) $${\text{log}}_{10}Pp=A-\frac{B}{C+T}$$

where *A,B,C* are constants connected with the concentration of the HF in the solution, *T* is the absolute temperature and *Pp* is the partial pressure of the resulting vapor. The values of the three constants can be found in several places, such as at NIST [23], and can be computed from partial pressure experimental measurements [24].

In the right part of Figure 9 are two FESEM images of a fiber surface before and after exposure to the HF vapors. After deposition and before the exposure, the surface appears smooth and covered by a thin continuous layer of a SiO_2_-like film. After the exposure, the surface becomes corroded as SiO_2_ is transformed into SiF_4_.

**Figure 10.**
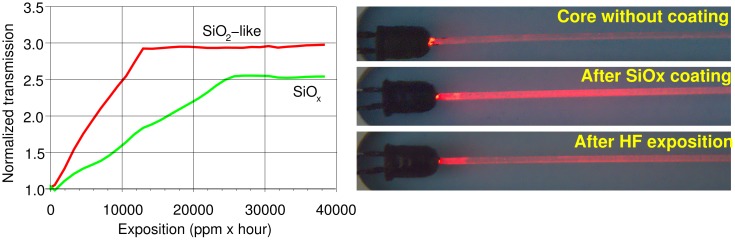
(**Left**) Fiber transmittance change with exposure to HF vapors. (**Right**) Example pictures of the same fiber in three different stages: (**top**) the fiber after cladding removal, but before the SiO_x_ deposition; (**middle**) the fiber after the SiO_x_ deposition; (**bottom**) the fiber after exposure to the HF vapors.

Figure 10 shows the fiber transmittance change due to the exposure to HF vapors. The left part of the figure shows two traces referring to two fibers obtained by using the two different discharge gas compositions. Both compositions lead to a thin coating, which is attacked by the fluoride ions, but the layer obtained in the presence of oxygen, and thus much less organic, results in an increased sensitivity that changes from less than 0.01%/(ppm · hour), in the case of the sensor obtained without oxygen, to about 0.02%/(ppm · hour) for the sensor obtained with oxygen presence, thus doubling the sensor sensitivity.

In the right part of Figure 10, three pictures of a fiber lit by a red LED in a dark field are reported. The three pictures refer to the same fiber in three different stages: At the top, the fiber after cladding removal, but before the SiO_x_ deposition. The light is almost completely confined inside the fiber and transmitted to the end. In the middle, the fiber after the SiO_x_ deposition. A remarkable part of the light is no longer guided by the fiber, so that only a minimal part arrives at the end. At the bottom, the fiber after exposure to the HF vapors. The SiO_x_ coating is almost completely destroyed, so that the fiber regains most of its capability to transmit the light.

## 5. Conclusions

Plastic optical fibers can be used as the key element for developing very low-cost sensors for environmental monitoring by removing the fiber cladding and by coating the core with a thin layer of a substance capable of reacting with the substance to be detected.

Sensors based on the measurement of the fiber transmittance change, even though less accurate than solutions based, for example, on fiber Bragg grating, are extremely inexpensive, have very low energy consumption and, in addition, do not require bulky interrogation systems. These advantages make them specifically suited for installation in adverse conditions and when cabling is not conceivable.

In addition to conventional sensors, which are sensitive to the actual value of the quantity of interest, the POF sensor can be easily designed to be sensitive to the total exposure of a quantity, *i.e.*, to the integral over time of that quantity.

Designing sensors with this behavior requires selecting a coating that exploits a non-reversible chemical reaction with the quantity of interest, but that provides interesting advantages over other conventional techniques based on post processing of the sensor outputs.

In fact, since the cumulative effect is obtained at the physical level, it is possible to avoid all of the problems connected with the presence of offsets, which would greatly impair an integration procedure obtained by post processing the sensor output, and permits detecting and measuring extremely low concentrations of the quantity of interest.

As an example, tests performed on a silver-coated fiber for the detection of H_2_S vapors in the cultural heritage field showed the possibility of arranging sensors with a sulfide sensitivity better than 0.2 ppb, *i.e.*, with the capability of monitoring the sulfide concentration in clean air. The described sensor showed an output change of about 0.3%/(ppb · hour), with a useful measuring range of 100 ppb · hour. This means that it is possible to arrange sensors, whose cost is of the order of $1, that can be used to measure the exposure to sulfide vapors in clean air for about one month.

Other tests performed on glass-like coated fibers for the detection of HF vapors for high energy applications proved the possibility of arranging sensors with an output sensitivity of 0.02%/(ppm · hour) and a measuring range of 15, 000 ppm · hour.

The good performance and high sensitivity of the these sensors encourage the development of sensing devices for other applications simply by changing the nature of the substance deposed on the fiber core.
